# Draft genome sequences of two wide-host-range phages of *Listeria monocytogenes* from food processing environments in the United States

**DOI:** 10.1128/mra.00358-24

**Published:** 2024-06-25

**Authors:** Phillip Brown, Samuel Kilcher, Jae-Won Kim, Martin Loessner, Sophia Kathariou

**Affiliations:** 1 Department of Plant and Microbial Biology, North Carolina State University, Raleigh, North Carolina, USA; 2 Laboratory of Food Microbiology, Institute of Food, Nutrition and Health, ETH Zurich, Zürich, Switzerland; 3 Department of Food, Bioprocessing and Nutrition Sciences, North Carolina State University, Raleigh, North Carolina, USA; Queens College Department of Biology, Queens, New York, USA

**Keywords:** *Listeria*, bacteriophages, food-borne pathogens, genomes, intracellular pathogens

## Abstract

*Listeria monocytogenes* is notorious for persistence in food facilities. Phages can significantly impact the ecology of *Listeria*, but there is a dearth of genome sequence data for *Listeria* phages from food processing ecosystems. We report the genome sequences of two *Listeria* phages from turkey processing facilities in the USA.

## ANNOUNCEMENT

*Listeria monocytogenes* is notorious for persistence in food processing environments (FPEs) and subsequent food contamination leading to severe disease (listeriosis) (1–3). Virulent, wide-host-range (WHR) phages can significantly impact the ecology and population dynamics of *L. monocytogenes* in FPEs, but most investigated WHR phages have been from farms and sewage (4–6). We previously isolated WHR phages from two turkey processing plants in the USA and showed that *L. monocytogenes* of serotypes 1/2a and 1/2b were frequently resistant to these phages (7). Here, we report the draft genome sequences of two of these phages, 20422-1 and 805405-1 (Table 1). Both phages were members of family *Herelleviridae*, genus *Pecentumvirus*. They had identical size (134 kb) and 3,347 bp direct terminal redundancies, and almost identical sequences (100% coverage, 99.96% nt identity) highly similar to the WHR phages A511 (GCF_000871125.1) and P100 (DQ004855.1) (97% coverage, approx. 98.4% nt identity) (genus *Pecentumvirus*) isolated in 1990 and 1997 from sewage in Germany (8, 9). The high genomic similarity of 20422-1 and 805405-1 raises the possibility that they represent different isolates of a WHR listeria phage disseminated in different turkey processing plants in the USA.

**TABLE 1:** Bacteriophages investigated in this study

Characteristics	20422-1	805405-1
Source	Food processing environment, conveyor belt, USA, Plant A	Food processing environment, floor drain, USA, Plant B
Isolation date	June 2004	May 2005
Family	*Herelleviridae*	*Herelleviridae*
Genus	*Pecentumvirus*	*Pecentumvirus*
Species	nA^*a*^	nA^*a*^
No. of contigs	1	1
Total length (bp)	134,755	134,754
N50 (bp)	134,755	134,754
Coverage (X)	6,742	10,832
No. of reads	6,056,756	9,731,674
GC content	35.9%	36.0%
Sequencing method	Illumina NovaSeq6000	Illumina NovaSeq6000
Read quality control tools	CLC Genomics Workbench version 2.0	CLC Genomics Workbench version 2.0
GenBank accession	PP101685.1	PP101686.1
BioSample accession	SAMN38501085	SAMN38501086
Sequence read archive accession	SRR26988306	SRR26988305

^*a*^ nA; Not available.

Bacteriophages were isolated as described (7) and propagated using the soft-agar overlay method with 1/2 brain-heart infusion (Biolife, Milan, Italy) as bottom agar (1% agar) and LC as top agar (0.4% lysogeny broth agar with 10mM CaCI_2_, 2mM MgSO_4_, and 10g/L glucose). Briefly, 200 µL log-phase host bacteria (*Listeria ivanovii* WSLC 3009, OD [Optical Density]_600_ = 0.5) were mixed with 10 µL phage dilution adjusted for semi-confluent lysis in 5 mL liquid top agar at 47°C and overlaid on the bottom agar. Plates were incubated at 30°C. Progeny virions were extracted from 40 semi-confluent plates using 5 mL SM buffer/plate (100mM NaCl, 8mM MgSO_4_, 50mM Tris, pH 7.4) and filter-sterilized (0.2 µm) to obtain lysates. Lysates were digested with DNase I (10 µg/mL) and RNase A (1 U/10  mL, 37°C, 30 min), and phages concentrated by polyethylene glycol (PEG) precipitation (7% PEG 8000 and 1 M NaCl) at 4°C for 16 hours. Phages were resuspended in 5 mL SM buffer, purified by CsCl isopycnic centrifugation (10), and dialyzed twice against 1,000× excess SM buffer. CsCl-purified phages were digested with proteinase K (200 µg/mL, 55°C, 30 min, in SM buffer with 10 mM EDTA, pH 8.0), and phage DNA was purified with two additional purification steps using phenol:chloroform:isoamyl alcohol (25:24:1) followed by DNA precipitation in ethanol as described (11). Library preparation and Illumina sequencing (2 × 150  bp, 5M reads) of purified DNA was performed by Eurofins Genomics Europe Sequencing GmbH (Constance, Germany). Sequence analysis and quality control utilized CLC Genomics version 2.0 (QIAGEN Bioinformatics). Adapter trimming was not required. Single contigs were obtained for both genomes by *de novo* assembly using the CLC Genomics Workbench. Terminal redundancies were identified by mapping Illumina reads to the unit genome, revealing a characteristic duplication of sequencing coverage over a 3,347 nt region. Coding DNA sequence identification and annotation was performed using the RAST server (12). Default parameters were used for all software unless otherwise specified.

## Data Availability

This Whole Genome Shotgun project has been deposited in NCBI under BioProject PRJNA1046509. The version described in this paper is the first version.
